# Comparative effects of 1.25 vs. 1.5 mmol/L dialysate calcium concentrations on blood pressure variability during hemodialysis: a two-center retrospective study

**DOI:** 10.3389/fmed.2026.1792552

**Published:** 2026-03-12

**Authors:** Daqing Hong, Changlian Li, Yun Zhang, Yu Liu, Zhangsuo Liu

**Affiliations:** 1 Department of Nephrology, The First Affiliated Hospital of Zhengzhou University, Zhengzhou, China; 2 Department of Nephrology, Sichuan Provincial People’s Hospital, Chengdu, China; 3 School of Medicine, University of Electronic Science and Technology of China, Chengdu, China; 4 Department of Nephrology, Affiliated Hospital, Southwest Medical University, Luzhou, China; 5 Department of Nephrology, The Second People’s Hospital of Neijiang, Neijiang, China; 6 Knowledge and Data Engineering Laboratory of Chinese Medicine, School of Information and Software Engineering, University of Electronic Science and Technology of China, Chengdu, China; 7 Department of Integrated Traditional and Western Nephrology, The First Affiliated Hospital of Zhengzhou University, Zhengzhou, China; 8 Henan Provence Research Center for Kidney Disease, The First Affiliated Hospital of Zhengzhou University, Zhengzhou, China

**Keywords:** blood pressure variability, calcium concentration, calcium flux, dialysate calcium, hemodialysis

## Abstract

**Introduction:**

Hemodialysis patients frequently experience blood pressure fluctuations, which are independently associated with adverse cardiovascular outcomes. However, the optimal dialysate calcium concentration for minimizing blood pressure variability (BPV) remains controversial.

**Methods:**

This retrospective cohort study investigated the differential effects of dialysate calcium concentrations of 1.25 mmol/L (DCa 1.25) versus 1.5 mmol/L (DCa 1.5) on intradialytic BPV. We analyzed 2,061 hemodialysis sessions from 711 maintenance hemodialysis patients across two centers in China. Patients were categorized based on dialysate calcium concentration: 1.25 mmol/L (*n* = 473 patients, 1,425 sessions) and 1.5 mmol/L (*n* = 238 patients, 636 sessions). BPV was defined as the difference between maximum and minimum blood pressure during each session.

**Results:**

Compared with DCa 1.5, group DCa 1.25 demonstrated significantly lower systolic blood pressure variability (SBPV) (25.23 ± 14.55 vs. 32.42 ± 17.71 mmHg, *p* < 0.001), diastolic blood pressure variability (DBPV) (9.78 ± 8.07 vs. 11.56 ± 9.67 mmHg, *p* < 0.001), and mean arterial pressure variability (MAPV) (15.41 ± 9.40 vs. 18.71 ± 11.10 mmHg, *p* < 0.001). Serum total calcium changes during dialysis were smaller in the 1.25 mmol/L group [0.04 (−0.06, 0.16) vs. 0.15 (0.04, 0.26) mmol/L, *p* < 0.001] and positively correlated with SBPV and MAPV. In multivariate generalized linear models adjusted for potential confounders, dialysate calcium concentration of 1.5 mmol/L was independently associated with greater BPV.

**Discussion:**

These findings suggest that lower dialysate calcium concentration may improve hemodynamic stability during hemodialysis by minimizing calcium flux and associated blood pressure fluctuations.

## Introduction

Blood pressure fluctuations during hemodialysis represent a critical clinical challenge, with intradialytic hypotension occurring in 10–70% and hypertension in 15–40% of HD patients, respectively (1–8). Importantly, these episodes demonstrate independent associations with heightened cardiovascular events and mortality risk (3, 7, 9). Furthermore, even in the absence of such acute events, intradialytic blood pressure variability (BPV), particularly systolic blood pressure (SBP) fluctuations, may contribute to an elevated risk of cognitive dysfunction (10), cardio-cerebrovascular events (11, 12), and even increased mortality (13, 14). Intradialytic blood pressure alterations are influenced by multiple factors, including patients’ disease status, interdialytic weight gain, blood pressure, and dialysis treatment parameters such as dialysate electrolyte concentrations, including dialysate calcium concentration (DCa) (15, 16). Calcium influences blood pressure through alterations in systemic vascular resistance, cardiac output or both.

DCa in clinical practice has evolved from initially adopting high DCa dialysis based on the widespread belief that dialysis patients generally suffer from hypocalcemia, to recognizing the harmful effects of positive calcium balance during dialysis and consequently leaning toward lower DCa, individualized approaches (17), or utilizing calcium profiling in clinical practice (18). However, to date, there remains insufficient evidence to recommend an optimal DCa (19).

Higher DCa may augment cardiac output, maintain vascular resistance, and reduce intradialytic blood pressure decreases (18, 20, 21), however a DCa of 1.75 mmol/L has been associated with increased vascular calcifications and arterial stiffness, worsening left ventricular function and was identified as a significant risk factor for all-cause mortality (22–28). On the contrary, DCa levels 1.25 mmol/L or lower may reduce intradialytic hypertension (29) but are associated with a higher occurrence of hypotensive episodes (21) and sudden cardiac death (30, 31), a phenomenon likely mediated by QT interval prolongation (32, 33). The relationship between DCa and intradialytic BPV is complex. Research indicates that high DCa (1.75 mmol/L) are associated with positive mean blood pressure variations [4.0 (−6.0, 12.2 mmHg)], while low DCa (1.25 mmol/L) correlate with negative variations [−3.2 (−9.8, 1.3 mmHg)] (22). Furthermore, high dialysate calcium concentrations may increase sympathetic stimulus during hemodialysis, potentially contributing to cardiovascular instability (22).

Despite growing recognition of dialysate calcium’s impact on cardiovascular outcomes during hemodialysis, substantial evidence gaps persist regarding the optimal DCa for minimizing BPV—a clinically significant determinant of patient prognosis. While DCa of 1.25 and 1.50 mmol/L represent the most widely prescribed regimens in contemporary clinical practice, there remains a striking paucity of comparative data examining their differential effects on BPV and related clinical outcomes. This methodological gap is further compounded by divergent guideline recommendations, with authoritative bodies offering conflicting guidance on optimal DCa selection (34, 35). Against this backdrop of clinical uncertainty and limited evidence, our study endeavors to address this critical knowledge void by comprehensively investigating the comparative effects of DCa 1.25 versus 1.50 mmol/L on BPV during hemodialysis sessions in a two-centered cohort of Chinese patients.

## Methods

### Study design and population

This retrospective cohort study analyzed electronic medical records from Sichuan Provincial People’s Hospital and The Second People’s Hospital of Neijiang between January 1, 2023, and December 31, 2023. The study included adult maintenance hemodialysis (MHD) patients aged ≥18 years who underwent regular hemodialysis treatment (≥2 sessions per week) during the study period. Patients with acute disease resulting in hospitalization, or incomplete blood pressure recordings were excluded.

Sessions with hemodialysis modalities with a DCa of 1.25 mmol/L or 1.5 mmol/L were included. Dialysate preparation for hemodialysis using commercially prepared acid concentrate and bicarbonate powder involves precise formulation to achieve target calcium concentrations of 1.25 mmol/L and 1.5 mmol/L. Dialyzers featuring polysulfone or polyethersulfone membranes were used in dialysis sessions. Patients were grouped according to dialysis calcium concentration: DCa 1.25 mmol/L group and DCa 1.5 mmol/L group.

### Data collection

Demographic data including age, gender, and primary renal disease were extracted from the database of hemodialysis software (Xizhizhu, Jiangsu Huibang Information Technology Company Limited, Xuzhou, Jiangsu, China). Dialysis parameters including dialysate calcium concentration, ultrafiltration volume (UF), body weight (pre-dialysis and post-dialysis), blood flow, dialysis duration, and blood pressure measurements (pre-dialysis, hourly during dialysis, and post-dialysis) were collected. Laboratory parameters including serum calcium (Ca), sodium (Na), potassium (K) magnesium (Mg), and hemoglobin (Hb), and albumin levels (ALB) from routine pre-dialysis blood tests and serum calcium from routine post were obtained. Dialysis adequacy indexes, including Kt/V (Clearance × Time / Volume of urea distribution) and Urea Reduction Ratio (URR), were automatically calculated within the hemodialysis software. Kt/V was calculated using single-pool Kt/V (spKt/V) with the Daugirdas second-generation equation:


spKt/V=−ln(R−0.008×t)+(4–3.5×R)×UF/W.

(Where: *R* = ratio of post-dialysis to pre-dialysis blood urea nitrogen concentration, t = dialysis session duration in hours, UF = ultrafiltration volume in liters, W = post-dialysis body weight in kilograms)


URR=100%×(1−C1/C0).

(Where: C0 = pre-dialysis blood urea nitrogen concentration, C1 = post-dialysis blood urea nitrogen concentration)

**Figure 1:**
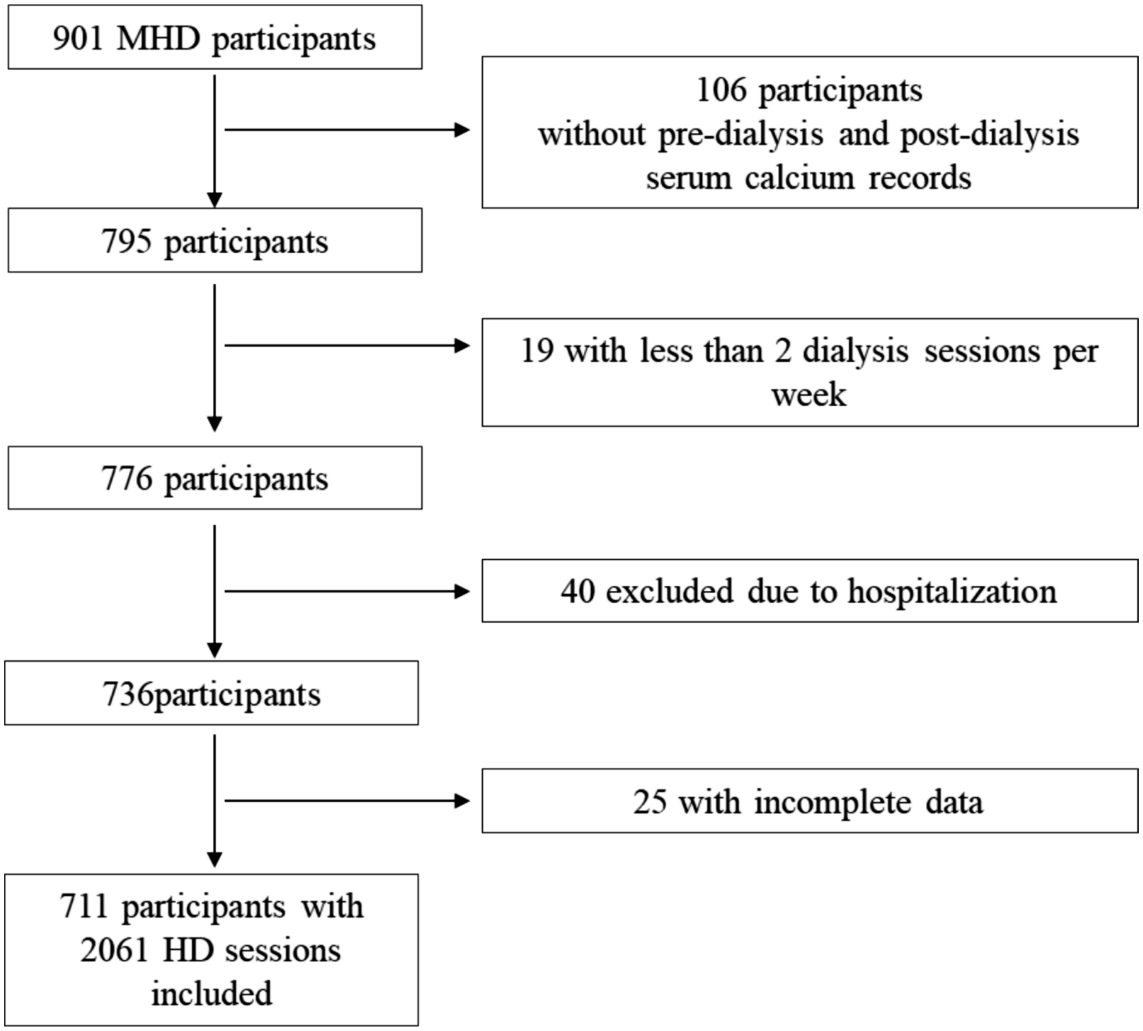
Flowchart of inclusion. MHD, maintenance hemodialysis; HD, hemodialysis.

This study was approved by the Clinical Ethics Review Committee of Sichuan Provincial People’s Hospital, The Medical Ethics Committee of Second People’s Hospital of Neijiang and adhered to the Declaration of Helsinki. All data were de-identified to ensure patient confidentiality, and the study strictly adhered to applicable laws and regulations governing data privacy and protection (Figure 1).

### Definitions

Mean arterial pressure (MAP) was calculated by adding the systolic blood pressure to twice the diastolic blood pressure (DBPV), then dividing the result by three. BPV was defined as the difference between maximum and minimum SBP recorded during each dialysis session, then SBP variability (SBPV), DBP variability (DBPV) and MAP variability (MAPV) were calculated. Change of serum total calcium (CCa) was defined as a difference between post-dialysis and pre-dialysis (post-dialysis minus pre-dialysis serum total calcium).

### Statistical analysis

Continuous variables were expressed as mean (standard deviation) or median (interquartile range) based on distribution. Categorical variables were expressed as frequencies and percentages. Comparisons between DCa 1.25 and DCa 1.5 were performed using t-tests, Mann–Whitney U tests, or chi-square tests as appropriate. Scatter plots and correlation analyses were utilized to examine: (1) the relationship between pre-dialysis serum total calcium and its changes during hemodialysis; and (2) the association between serum total calcium changes and intradialytic BPV. Furthermore, we conducted linear regression analyses to model the relationship between pre-dialysis serum total calcium concentrations and CCa during dialysis sessions. Separate linear regression models were fitted for the DCa 1.25 and DCa 1.5 groups, from which we derived and compared the coefficient of determination (R^2^), regression coefficients (slopes), and the pre-dialysis serum calcium concentration corresponding to zero net CCa (x-intercept). Generalized linear model (GLM) was used to identify independent factors associated with BPV and DCa after adjustment for potential confounders. To further validate the reliability of the results, a sensitivity analysis was carried out using propensity score matching (PSM). A 1:1 nearest neighbour matching without replacement was performed under the constraint that maximum standardized mean difference between the groups had to be < 0.1 for all confounders. Following PSM, between-group blood pressure variability was re-analyzed to validate the reliability of the findings. Statistical analyses were performed using IMB SPSS Statistics (version 27).

## Results

### Patient characteristics

A total of 901 participants were screened, and 711 patients with 2,061 hemodialysis sessions were included in the analysis. Group DCa 1.25 comprised 473 patients with 1,425 dialysis sessions, while group DCa 1.5 included 238 patients with 636 sessions. Baseline characteristics showed that age and primary renal disease composition was similar between the two groups, with group DCa 1.25 having a higher proportion of male patients compared to group DCa 1.5 (Table 1).

**Table 1:** Demographics and clinical characteristics of study population.

Variable	DCa1.25 (*n* = 473)	DCa1.5 (*n* = 238)	*P*
Age (years)	59.97 (14.09)	59.59 (14.32)	0.734
Male (*n*, %)	289 (61.1%)	115 (48.3%)	0.001
Primary disease (*n*, %)			0.555
DKD	93 (19.7%)	49 (20.6%)	
GN	73 (15.4%)	30 (12.6%)	
PKD	14 (3.0%)	7 (2.9%)	
Others	56 (11.8%)	21 (8.8%)	
Unknown	237 (50.1%)	131 (55.0%)	

DKD, diabetic kidney disease; PKD, polycystic kidney disease; GN, glomerulonephritis.

### Pre-dialysis and post-dialysis parameters

Pre-dialysis Hb was similar in two groups. Pre-dialysis parameters including ALB, pre-dialysis Ca, Mg, and body weight were higher, while Na and K were lower in group DCa 1.25 than DCa 1.5. Post-dialysis parameters including Ca, Na and K were lower while body weight was higher in group DCa 1.25 than DCa 1.5. UF, Kt/V and URR was lower, while blood flow and session time was higher in group DCa1.25 than DCa 1.5 (Table 2).

**Table 2:** Pre-dialysis and post-dialysis laboratory and treatment parameters.

Variable	DCa1.25 (*n* = 1425)	DCa1.5 (*n* = 636)	*p*
Hb (g/L)	111.76 (16.73)	112.58 (17.99)	0.330
ALB (g/L)	41.20 (3.73)	39.12 (3.28)	<0.001
Ca (mmol/L)			
Pre-dialysis	2.27 (0.23)	2.19 (0.20)	<0.001
Post-dialysis	2.33 (0.16)	2.34 (0.14)	0.016
Na (mmol/L)			
Pre-dialysis	137.90 (3.20)	139.08 (3.14)	<0.001
Post-dialysis	138.55 (2.76)	140.14 (2.32)	<0.001
Mg (mmol/L)	1.13 (0.17)	1.05 (0.23)	<0.001
K (mmol/L)			
Pre-dialysis	4.74 (0.79)	4.84 (0.74)	0.005
Post-dialysis	3.42 (0.47)	3.54 (0.42)	<0.001
Body weight (kg)			
Pre-dialysis	61.23 (11.82)	59.05 (11.68)	<0.001
Post-dialysis	59.35 (11.56)	57.02 (11.33)	<0.001
UF (kg)	1.90 (0.91)	2.01 (0.76)	0.002
Kt/V	1.44 (0.31)	1.47 (0.29)	0.034
URR (%)	68.59 (19.37)	70.49 (8.45)	0.002
Blood flow (ml/min)	242.90 (49.06)	231.24 (24.05)	<0.001
Session time (min)	235.75 (14.60)	231.21 (19.94)	<0.001

Hb, hemoglobin; ALB, serum albumin; Ca, serum calcium; Na, serum sodium; Mg, serum magnesium; K, serum potassium; UF, ultrafiltration volume; Kt/V, Clearance × Time / Volume of urea distribution; URR, urea reduction ratio.

### Blood pressure variability during hemodialysis sessions

Patients receiving DCa 1.25 exhibited significantly lower pre-dialysis, post-dialysis, maximum, and minimum values for SBP, DBP, and MAP compared with those receiving DCa 1.5 (Table 3). This resulted in markedly reduced BPV indices in the DCa 1.25 group: SBPV (25.23 ± 14.55 vs. 32.42 ± 17.71 mmHg, *p* < 0.001), DBPV (9.78 ± 8.07 vs. 11.56 ± 9.67 mmHg, *p* < 0.001), and MAPV (15.41 ± 9.40 vs. 18.71 ± 11.10 mmHg, *p* < 0.001).

**Table 3:** Blood pressure variability indices during hemodialysis sessions.

Variable	Group DCa1.25		Group DCa1.5 (*n* = 636)		p	p
	Before matching *n* = 1425	After matching *n* = 438	Before matching *n* = 636	After matching *n* = 438	Before matching	After matching
CCa (mmol/L)	0.04 (−0.06, 0.16)	0.04 (−0.05,0.16)	0.15 (0.04,0.26)	0.14 (0.03,0.25)	<0.001	<0.001
Pulse (beat/min)						
Pre-dialysis	76.36 (11.47)	78.50 (10.94)	83.20 (12.18)	79.18 (12.11)	<0.001	0.384
Post-dialysis	76.89 (12.75)	76.76 (11.72)	79.01 (11.73)	78.25 (11.66)	<0.001	0.059
SBP (mmHg)						
Pre-dialysis	141.71 (21.11)	145.78 (21.36)	149.07 (20.30)	144.47 (14.70)	<0.001	0.341
Post-dialysis	131.12 (19.92)	133.65 (19.69)	135.83 (20.86)	133.93 (21.04)	<0.001	0.837
Maximum	147.68 (20.10)	151.22 (20.20)	156.56 (19.48)	155.83 (18.60)	<0.001	<0.001
Minimum	121.54 (18.64)	124.22 (18.89)	124.14 (19.74)	122.18 (18.59)	0.004	0.107
SBPV	25.23 (14.55)	27.01 (15.26)	32.42 (17.71)	33.66 (15.29)	<0.001	<0.001
DBP (mmHg)						
Pre-dialysis	75.50 (13.80)	78.02 (13.12)	79.82 (15.51)	77.26 (14.70)	<0.001	0.417
Post-dialysis	73.00 (12.93)	74.17 (12.46)	79.97 (13.45)	78.85 (12.80)	<0.001	<0.001
Maximum	78.42 (13.34)	80.33 (12.72)	84.70 (14.89)	84.28 (14.08)	<0.001	<0.001
Minimum	68.39 (12.73)	70.12 (12.43)	72.84 (13.68)	71.48 (12.90)	<0.001	0.113
DBPV	9.78 (8.07)	10.22 (8.78)	11.56 (9.67)	12.80 (9.71)	<0.001	<0.001
MAP (mmHg)						
Pre-dialysis	97.57 (13.95)	100.61 (13.45)	102.90 (14.72)	99.66 (13.83)	<0.001	0.305
Post-dialysis	92.40 (13.49)	94.00 (13.05)	98.59 (14.09)	97.21 (13.75)	<0.001	0.373
Maximum	101.51 (13.38)	103.96 (12.76)	108.65 (14.09)	108.13 (13.25)	<0.001	<0.001
Minimum	86.10 (13.06)	88.15 (13.00)	89.94 (13.83)	88.38 (13.30)	<0.001	0.797
MAPV	15.41 (9.40)	15.81 (9.55)	18.71 (11.10)	19.75 (9.72)	<0.001	<0.001

CCa, change of serum total calcium; SBP, systolic blood pressure; SBPV, systolic blood pressure variability; DBP, diastolic blood pressure; DBPV, diastolic blood pressure variability; MAP, mean arterial pressure; MAPV, mean arterial pressure variability.

The independent associations between DCa and BPV indices were demonstrated across three separate GLM, each adjusting for comprehensive confounding variables including demographic factors (gender, age), dialysis parameters (duration, blood flow rate, UF, post-dialysis weight, URR, Kt/V), biochemical markers (serum Ca, Na, K, Mg, Hb, ALB), and relevant pre-dialysis blood pressure measures. All models achieved statistical significance [Omnibus tests: χ^2^ (16) = 413.835, 121.841, and 240.899 respectively; all *p* < 0.001], with DCa independently predicting SBPV (*p* < 0.001), DBPV (*p* = 0.031), and MAPV (*p* < 0.001). To adjust for the imbalance in the distribution between groups, we performed PSM on the main indicators including gender, age, blood flow rate, UF, post-dialysis weight, URR, serum Ca, Na, K, Hb, ALB, pre-dialysis blood pressure measures, finally enrolling 438 pairs of dialysis cases. Through comparison, DCa [0.14 (0.03, 0.25) vs. 0.04 (−0.05, 0.16) mmol/L], SBPV (33.66 ± 15.29 vs. 27.01 ± 15.26 mmHg, *p* < 0.001), DBPV (12.80 ± 9.71 vs. 10.22 ± 8.78 mmHg, *p* < 0.001), and MAPV variability (19.75 ± 9.72 vs. 15.81 ± 9.55 mmHg, *p* < 0.001) in the DCa1.5 group were significantly higher than those in the DCa1.25 group (Table 3).

### Change of serum total calcium and blood pressure variability

**Figure 2:**
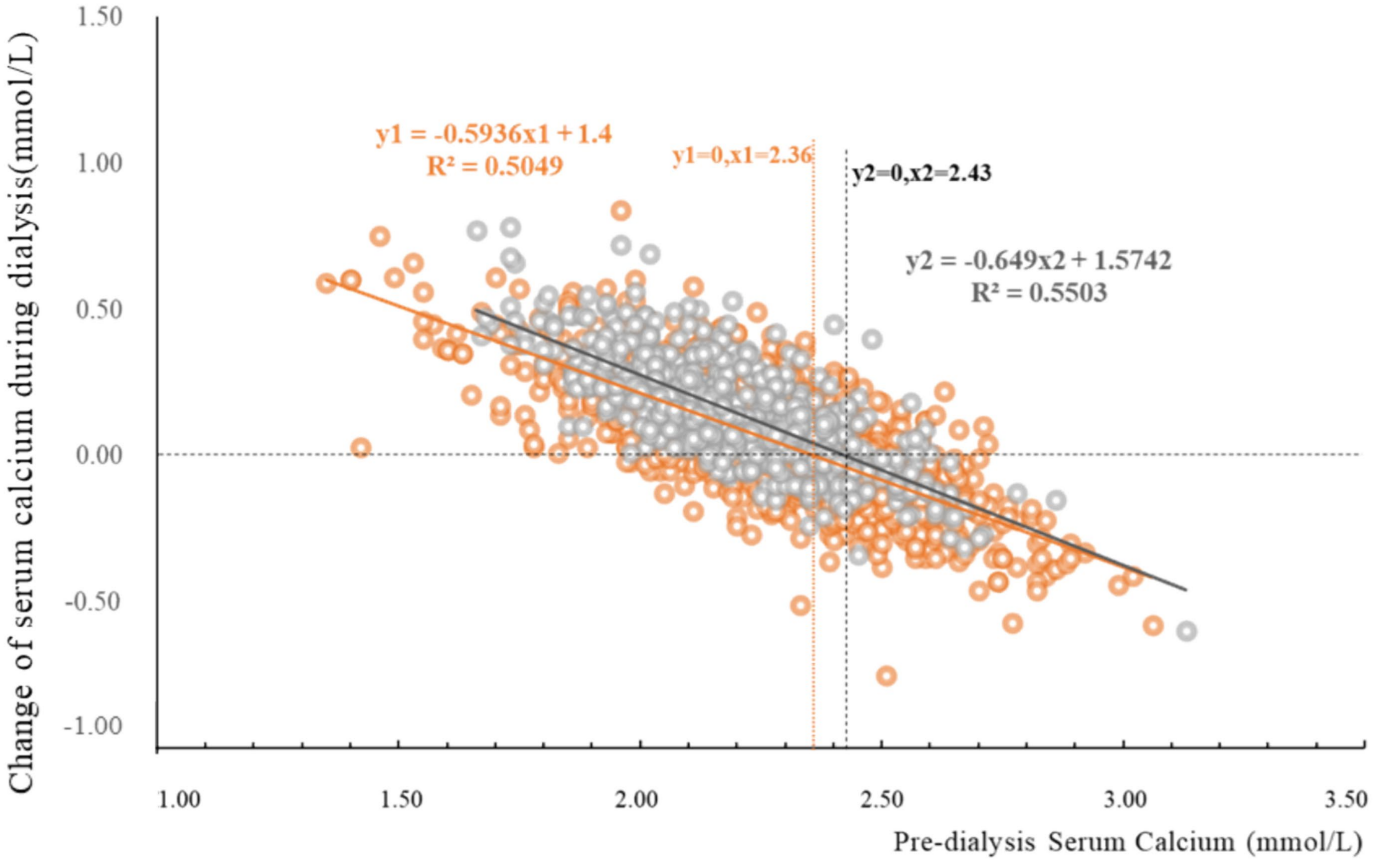
Change of serum total calcium and pre-dialysis serum total calcium. This figure illustrates the relationship between pre-dialysis serum total calcium (horizontal axis) and the change in post-dialysis serum total calcium (vertical axis, calculated as post-dialysis serum total calcium minus pre-dialysis serum total calcium). The orange circles represent group DCa 1.25, with the orange line showing the fitted linear correlation curve for this group. The orange line intersects the vertical axis at a pre-dialysis calcium of 2.36 mmol/L, and the orange equation along with R^2^ represents the correlation results for this curve. The gray circles indicate group DCa 1.5, with the dark gray line depicting the fitted linear correlation curve for this group. The dark gray line intersects the vertical axis at a pre-dialysis calcium of 2.43 mmol/L, and the dark gray equation along with R^2^ represents the correlation results for this curve. Group DCa 1.5 demonstrates a more pronounced effect on total calcium changes before and after dialysis compared to group DCa 1.25.

The magnitude of CCa was significantly greater in group DCa 1.5 [0.15 (0.04, 0.26) mmol/L] compared to group DCa 1.25 [0.04 (−0.06, 0.16) mmol/L] (*p* < 0.001, Table 3). Linear regression analysis revealed significant correlations between CCa and pre-dialysis serum total calcium (Figure 2). The DCa 1.5 condition exhibited enhanced calcium transfer kinetics, evidenced by increased slope magnitude (−0.649 vs. − 0.594) and elevated calcium balance point (2.43 vs. 2.36 mmol/L), indicating amplified calcium flux dynamics at higher dialysate calcium concentrations.

**Figure 3:**
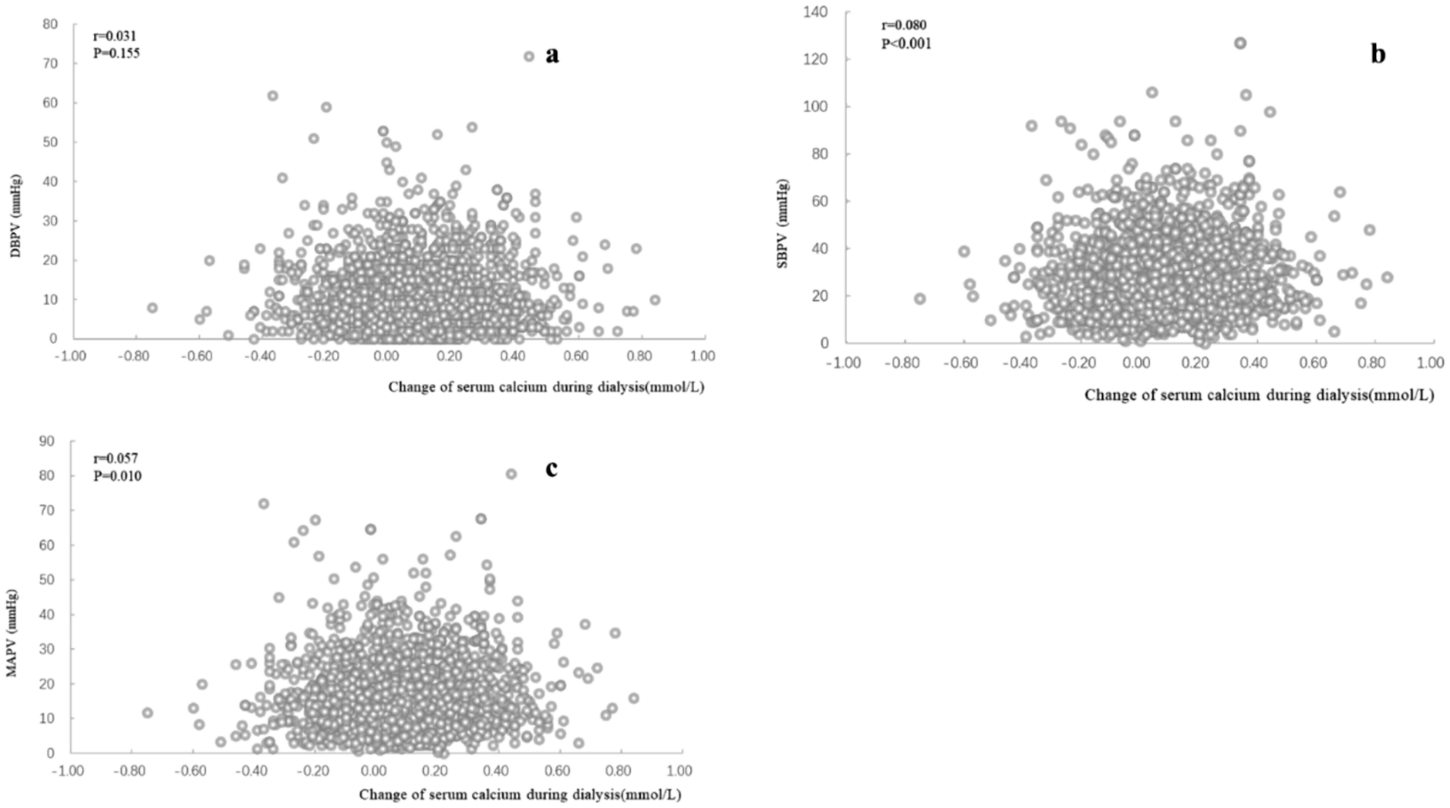
Blood pressure variabilities and change of serum total calcium and pre-dialysis serum total calcium. Figure showing the relationship between serum calcium changes (mmol/L, horizontal axis) and BPV (mmHg, vertical axis). The Pearson correlation coefficient is denoted by *r*. Subfigure **(a)** represents SBPV (systolic blood pressure variability), **(b)** represents DBPV (diastolic blood pressure variability), and **(c)** represents MAPV (mean arterial pressure variability).

CCa during hemodialysis showed a significant positive correlation with SBPV (*r* = 0.080, *p* < 0.001) and MAPV (0.057, *p* = 0.010) but not DBP (*r* = 0.031, *p* = 0.155) (Figure 3).

## Discussion

The relationship between dialysate calcium concentration and blood pressure during hemodialysis has been extensively investigated, with most studies focusing primarily on intradialytic hypertension or hypotension episodes (19, 21, 36, 37). However, BPV, which represents the magnitude of blood pressure fluctuations during dialysis sessions, has received relatively limited attention despite its clinical significance. Growing evidence indicates that increased BPV is independently associated with adverse outcomes including cardiovascular events and mortality in hemodialysis patients (10–14). Our study addresses this important knowledge gap by comprehensively examining the differential effects of commonly used dialysate calcium concentrations (1.25 vs. 1.5 mmol/L) on intradialytic BPV.

While traditional statistical measures such as standard deviation, coefficient of variation, and mean true variability provide valuable insights into blood pressure fluctuation patterns and are essential for comprehensive hemodynamic assessment, the peak-trough approach offers distinct clinical advantages in the dialysis setting. This methodology directly captures the most significant blood pressure fluctuations during dialysis sessions, enabling the detection of clinically relevant events such as intradialytic hypotension or hypertensive onsets. The peak-trough method provides greater intuitive clarity for clinical interpretation and facilitates immediate decision-making, particularly when healthcare providers perform repeat measurements to reduce measurement error upon observing unusually high or low blood pressure readings. Furthermore, this technique demonstrates heightened sensitivity to acute blood pressure changes within individual dialysis sessions. Importantly, in retrospective studies with hourly blood pressure measurements, there may be insufficient data points for robust statistical calculations, making the peak-trough approach particularly practical and meaningful. Research has demonstrated that absolute blood pressure differences are associated with mortality risk in dialysis patients (13, 38). Based on aforementioned reasons, we defined BPV as the difference between the highest and lowest blood pressure values during dialysis sessions.

According to this definition, we found that patients receiving dialysate calcium concentration of 1.5 mmol/L exhibited significantly greater blood pressure variability compared to those receiving 1.25 mmol/L. Specifically, we observed marked differences in systolic blood pressure variability (25.23 ± 14.55 vs. 32.42 ± 17.71 mmHg, *p* < 0.001), diastolic blood pressure variability (9.78 ± 8.07 vs. 11.56 ± 9.67 mmHg, *p* < 0.001), and mean arterial pressure variability (15.41 ± 9.40 vs. 18.71 ± 11.10 mmHg, *p* < 0.001) between the DCa 1.25 and DCa 1.5 groups, respectively. Significant baseline disparities were detected between treatment groups, notably lower serum albumin concentrations and pre-dialysis calcium levels in the DCa1.5 group compared to the DCa1.25 group, indicating potential confounding by indication in treatment choice. To mitigate this methodological limitation, we implemented comprehensive sensitivity analyses and found these associations remained statistically significant after comprehensive adjustment for potential confounding factors using generalized linear models, and sensitivity analysis using propensity score matching method. While the inter-group difference in SBPV was approximately only 7 mmHg, the DCa1.5 group exhibited a mean SBPV exceeding 30 mmHg. Emerging evidence indicates that SBPV values above this threshold, and even values as low as 20 mmHg, are associated with elevated mortality risk when compared to lower SBPV (13, 38), potentially indicating clinically meaningful implications for adverse outcomes. However, this observation warrants confirmation through additional prospective studies.

To investigate the underlying mechanisms contributing to these observed differences, we further examined the effects of different dialysate calcium concentrations on serum calcium changes during hemodialysis. Our findings revealed that the magnitude of serum total calcium changes was significantly greater in the DCa 1.5 group [0.15 (0.04, 0.26) mmol/L] compared to the DCa 1.25 group [0.04 (−0.06, 0.16) mmol/L] (*p* < 0.001). Notably, patients in the DCa 1.5 group experienced significant increases in post-dialysis serum calcium levels, whereas those in the DCa 1.25 group showed minimal changes, suggesting a more stable calcium balance with lower dialysate calcium concentrations. Furthermore, we identified significant positive correlations between serum calcium changes during dialysis and systolic blood pressure variability (*r* = 0.080, *p* < 0.001) and mean arterial pressure variability (*r* = 0.057, *p* = 0.010), but not with diastolic blood pressure variability (*r* = 0.031, *p* = 0.155).

These findings are consistent with previous research demonstrating the critical role of calcium in hemodynamic regulation during hemodialysis. Changes in serum ionized calcium levels have been shown to influence blood pressure through alterations in systemic vascular resistance, myocardial contractility and cardiac output (18, 20, 21, 39–41). Several studies have reported that higher dialysate calcium concentrations can enhance myocardial contractility and cardiac output and vascular tone, sympathetic stimulus and arterial stiffness but worsen left ventricular function (18, 22, 24, 26, 39, 40), potentially contributing to greater blood pressure fluctuations. Although the underlying mechanism for our findings is believed to involve alterations in serum calcium ion concentration, our study identifies an association between serum total calcium and intradialytic BPV. Research has demonstrated a strong linear correlation between serum ionized calcium and total calcium in hemodialysis patients (42). Given that intradialytic calcium ion flux is primarily driven by the concentration gradient between dialysate and blood, changes in serum total calcium may partially reflect variations in ionized calcium trends. Therefore, we hypothesize that the relationship between serum total calcium changes and intradialytic BPV may be mediated through ionized calcium fluctuations. However, due to limitations in ionized calcium testing accessibility and the retrospective nature of our study, we could not directly analyze whether alterations in dialysate calcium mediate blood pressure changes via serum ionized calcium concentration changes. Furthermore, we were unable to identify large-scale studies examining the relationship between pre-dialysis and post-dialysis ionized calcium concentration changes and blood pressure. Consequently, this finding should be interpreted with caution, and ionized calcium measurements remain the preferred method for clinical assessment when available.

The pathophysiological mechanisms underlying our observations likely involve complex interactions between calcium and cardiovascular function. Calcium ions play pivotal roles in the contractile processes of both vascular smooth muscle cells and cardiac myocytes (43, 44). Higher dialysate calcium concentrations may lead to increased systemic vascular resistance through enhanced calcium influx into vascular smooth muscle cells, while potentially increasing sympathetic nervous system activity, further contributing to hemodynamic instability (22, 23). Additionally, elevated calcium levels have been associated with increased myocardial contractility, which may contribute to greater blood pressure variability during dialysis sessions (39).

Our study makes several important contributions to the existing literature. First, we provide comprehensive evidence regarding the differential effects of two commonly used dialysate calcium concentrations on BPV, an outcome that has been underexplored despite its clinical relevance. Second, we elucidate the potential mechanisms linking dialysate calcium concentration to BPV through serum calcium changes, providing biological plausibility for our observations. Finally, our findings have important clinical implications, suggesting that individualization of dialysate calcium concentration may represent a modifiable factor for improving hemodynamic stability during hemodialysis.

However, several limitations of our study should be acknowledged. First, the retrospective design, coupled with discretionary dialysate calcium selection by clinicians, introduces potential selection bias and precludes causal inference despite statistical adjustments. Second, we did not measure ionized calcium concentrations, which may provide more physiologically relevant information regarding calcium balance during hemodialysis. Third, long-term clinical outcomes such as cardiovascular events and mortality were not assessed in our analysis. Fourth, the potential influence of medications affecting calcium metabolism or blood pressure on our findings requires further investigation. Finally, given the retrospective nature of our study, significant baseline imbalances were observed between groups, which, despite extensive statistical adjustment including multivariate regression models, may not completely eliminate the potential for residual confounding. Therefore, prospective, large-scale, multicenter randomized controlled trials are warranted to validate these findings.

Despite these limitations, our study provides valuable insights into the relationship between dialysate calcium concentration and blood pressure variability during hemodialysis. The reduction in BPV observed with lower dialysate calcium concentration (1.25 mmol/L) suggests that this approach may benefit patients prone to hemodynamic instability during dialysis sessions. The clinical significance of these findings is underscored by the established association between BPV and adverse outcomes in hemodialysis patients (10–13). Our results support the individualization of dialysate calcium prescription as an important strategy for optimizing hemodynamic stability in maintenance hemodialysis patients.

## Conclusion

In conclusion, our study demonstrates that compared with a dialysate calcium concentration of 1.5 mmol/L, a concentration of 1.25 mmol/L is associated with lower blood pressure variability during hemodialysis. This effect appears to be mediated, at least in part, by reduced serum calcium changes during dialysis with lower calcium concentrations. These findings have important clinical implications for the management of hemodialysis patients and support the individualization of dialysate calcium concentration as a potential strategy to improve hemodynamic stability. Future prospective studies are needed to confirm these findings and evaluate the long-term clinical outcomes associated with different dialysate calcium concentrations.

## Data Availability

The original contributions presented in the study are included in the article/supplementary material, further inquiries can be directed to the corresponding authors.
